# Commercial Cannabinoid Oil-Induced Stevens-Johnson Syndrome

**DOI:** 10.1155/2020/6760272

**Published:** 2020-02-19

**Authors:** Han Y. Yin, Nicholas Hadjokas, Kanish Mirchia, Robert Swan, Samuel Alpert

**Affiliations:** ^1^Department of Ophthalmology and Visual Sciences, SUNY Upstate Medical University, Syracuse, NY, USA; ^2^Herbert Wertheim College of Medicine, Florida International University, Miami, FL, USA; ^3^Department of Pathology, SUNY Upstate Medical University, Syracuse, NY, USA

## Abstract

**Purpose:**

To report an unusual presentation of commercial cannabidiol (CBD) oil-induced Stevens-Johnson Syndrome/toxic epidermal necrolysis (SJS-TEN).

**Methods:**

A 56-year-old woman presented with acute onset of a diffuse, blistering, maculopapular rash with over 30% total body surface area (BSA) involvement two days after taking CBD oil sublingually for chronic pain. Biopsy confirmed SJS-TEN. Ophthalmology was consulted and mild eye involvement was found. She was started on topical cyclosporine, prednisone, moxifloxacin, and erythromycin ointment to prevent progression, which was successful. She was otherwise treated with supportive therapy in the intensive care burn unit and ultimately passed away from septic shock.

**Conclusion:**

In this case, we described an unusual drug-induced SJS from a commercial, non-FDA-regulated cannabis product. The use of a commercial CBD product should be cautioned due to potential for series of drug reactions to the cannabis product and the risk for reaction to other unregulated other pharmacological components.

## 1. Background

Stevens-Johnson syndrome (SJS) and toxic epidermal necrolysis (TEN) are devastating mucocutaneous hypersensitivity reactions characterized by progressive severe exfoliative rash affecting mainly the skin and mucous membranes. Often, these are preceded by diffuse mucocutaneous tenderness and erythematous macules and blisters [1]. These two syndromes exist on a spectrum, with SJS involving less than 10% body surface area (BSA) and TEN involving greater than 30% BSA.

SJS-TEN have been reported in various age groups but occurs more frequently in females, HIV-infected patients, and the elderly. Common causes include medications including antibiotics and antiepileptics and infections such as mycoplasma; however, 50% of cases remain idiopathic [2]. The increased incidence in the elderly population is likely due to increased medication usage with age [3]. Drug hypersensitivity has been associated with genetic factors. In certain ethnic groups, medications like carbamazepine and allopurinol have a strong correlation with human leukocyte antigen- (HLA-) B^∗^1502 and HLA-B^∗^5801, respectively [4]. Unfortunately, the RegiSCAR study demonstrated that HLA-B^∗^1502 is not a confirmatory marker for any of the high-risk drugs known to cause SJS-TEN in Europeans; so, these HLA markers cannot be used to confirm the diagnosis [2, 4].

Mortality rates for SJS-TEN range from 10 to 50%. Thus, prompt identification and discontinuation of the causative agent is vital. There have been reported cases of SJS from complementary and alternative products [5], but few from cannabis products. Cannabidiol (CBD) is one of the active ingredients of cannabis that stimulates cannabinoid receptors without causing psychotropic effects. It is currently being investigated for use in childhood epilepsy syndromes, anxiety, and chronic pain. Herein, we present an unusual case of drug-induced SJS from commercial CBD oil.

## 2. Case Report

A 56-year-old female with a past medical history of herniated disc with chronic pain, hypertension, and coronary artery disease presented to her local emergency room for diffuse vesicular rash and skin ulceration management. She denies prior history of dermatological rashes, or recent sick contacts, fever, or malaise prior to the onset of her symptoms. One week prior, she had tried a new liposomal CBD extract spray (Natural Native, Norman, Oklahoma, 73072) sublingually. Two days following the use of the new CBD product, she noticed a mild rash on her extremities, which was treated by her primary care physician with diphenhydramine and oral prednisone without improvement. Her symptoms progressed and she developed diffuse erythematous and vesicular rashes involving her entire body over the next 48 hours. She was transferred to a university hospital for a higher level of care. Her chronic outpatient medications for the past 5 years included famotidine, lisinopril-hydrochlorothiazide, and meloxicam. She had previously used other CBD products without any adverse effect.

**Figure 1:**
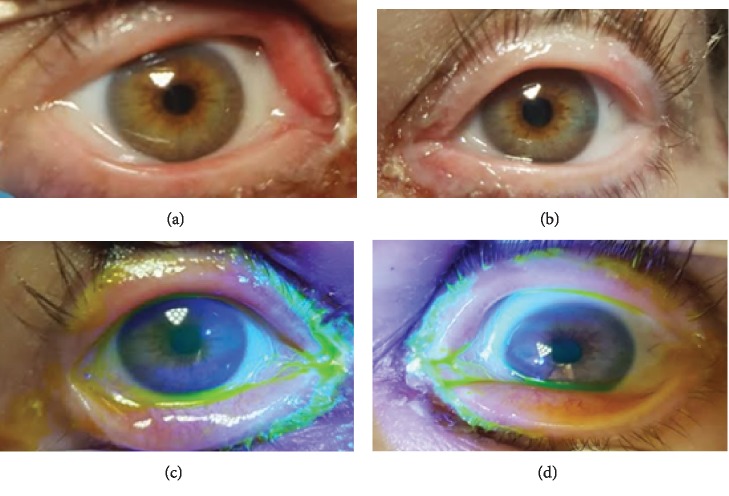
(a, b) External photo and (c, d) with fluorescein, without conjunctival injection, signs of pseudomembrane, or gross epithelial defect OU.

**Figure 2:**
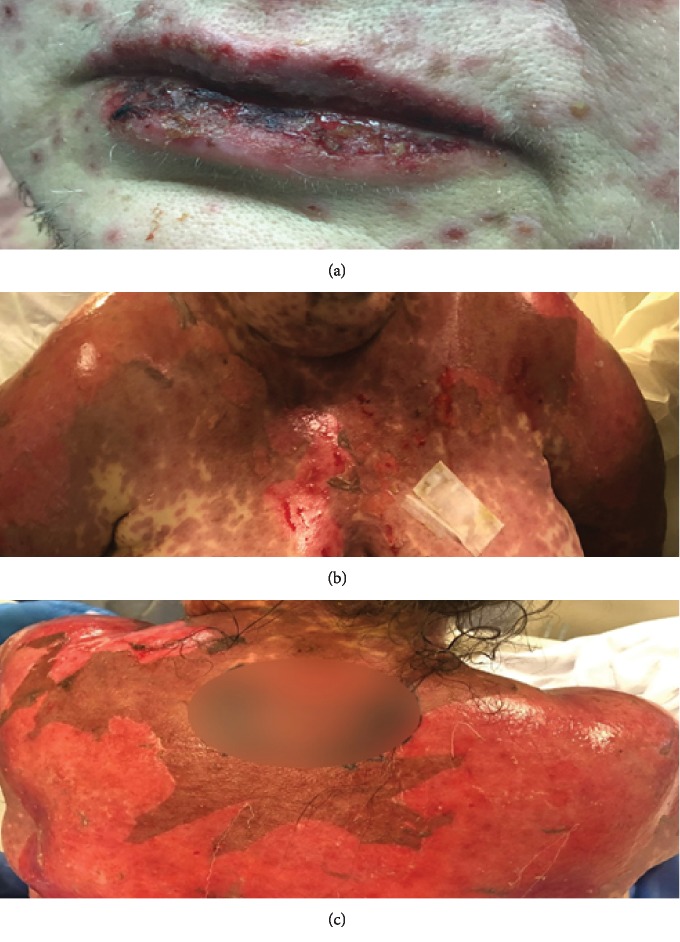
(a) External photo of diffuse oral ulceration and erythematous macules with vesicles on the trunk (b) and bullae and denudation on the back (c).

**Figure 3:**
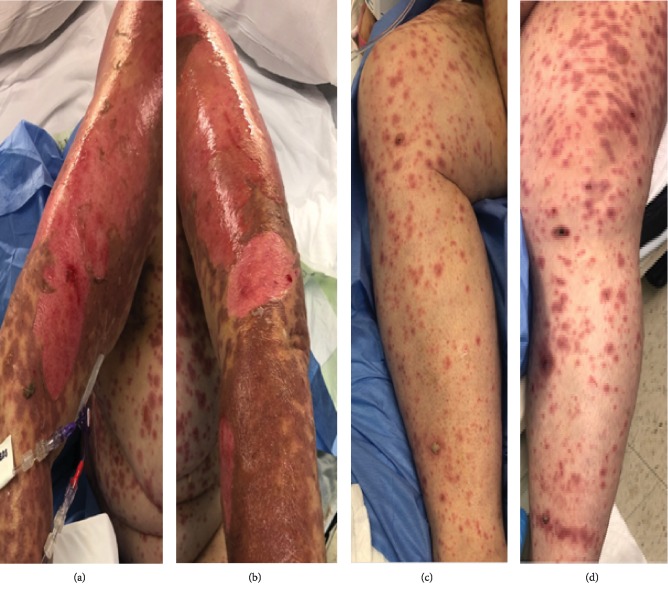
External photo of diffuse ruptured vesicle and ulceration right and left upper extremities (a, b) and erythematous macules with central necrosis on the right and left lower extremities (c, d).

On exam, she had diffuse erythematous macules and central necrosis with vesicles on her face. She had been experiencing crusting of the eyelashes and itching of the medial canthi, but she denied changes in vision and foreign body sensation. Her best corrected visual acuity was 20/20 with pinhole in both eyes, intraocular pressures were 16 in the right eye, 17 in the left eye, pupils were equal and briskly reactive without APD, and extraocular muscles were full. Her ophthalmic exam showed a maculopapular rash on the upper and lower eyelids without conjunctival injection, fibrin formation, or corneal epithelial defect in either eye (Figures 1(a)–1(d)). She had extensive oral mucosal ulceration (Figure 2(a)) and generalized erythematous macules and blisters with multiple ruptured bullae on her trunk and back (Figures 2(a) and 2(b)). In addition, she had extensive erythematous macules and central necrosis on all four extremities (Figures 3(a)–3(d)) along with urethral and labial involvement, totaling 30 percent BSA.

The patient was admitted to the burn intensive care service for presumed SJS-TEN and started on a wound care regimen and intravenous fluid. Her outpatient oral prednisone was discontinued in addition to all CBD products. She was started on topic cyclosporine drops OU.

**Figure 4:**
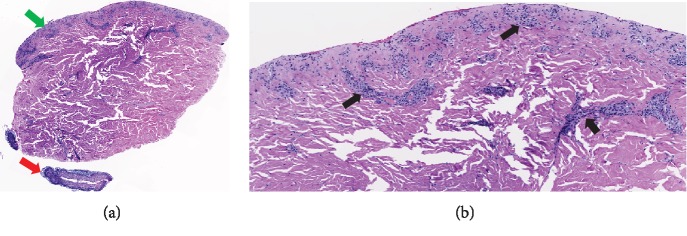
Biopsy of the skin from the patient. Biopsy examination under 20x magnification (a): green—subepidermal blister, red—detached and necrotic epidermis and under 100x magnification (b): black arrows detailing chronic inflammation.

BID, prednisolone OU QID for 1 week, and moxifloxacin OU QID for 1 week to prevent progression of ophthalmic involvement. Punch biopsy was obtained and was consistent with SJS (Figure 4). The patient expired about 1 month after initial presentation from septic shock.

## 3. Discussion

This case addresses some interesting considerations in the management and etiologies of SJS-TEN. Management usually consists of hospital admission, ideally with isolation in a burn unit to minimize potential hospital-acquired infections and provide supportive therapy including IV hydration for adequate nutrition. For drug-induced SJS-TENS, the offending agent is stopped immediately.

Medications for management of SJS-TEN at the acute stage are controversial. Some believe that T-cell-mediated immunologic responses are the cause of SJS-TEN and have consequentially administered high-dose glucocorticoids, cyclophosphamide, or cyclosporine as a means of arresting the progression of skin lesions [6]. These drugs, however, are of unproven benefit in the acute stage and may have systemic adverse effect when used at the chronic stage [7].

The use of systemic glucocorticoids is controversial. Many physicians no longer prescribe glucocorticoids, due to limited evidence to support their use. Others treat empirically under the assumption that such a short-term high dose of steroids can curb disease progression [6]. There is concern that systemic high-dose glucocorticoids may heighten the existing risk of inciting infections. Despite the associated risk with systemic steroids, some suggested that high dose of steroids (methylprednisolone 500 or 1000 mg/day for 3 to 4 days) within four days of disease onset has beneficial therapeutic effect in preventing ocular complications [8]. In addition, topical steroid also shows great promise for preventing corneal epithelial stem cell loss in the limbal region and cicatricial changes. Systemic therapeutic measures such as TNF-alpha inhibitor, thalidomide, and cyclophosphamide have been associated with increased mortality [9]. In our case, systemic steroids were not continued in the inpatient setting, due to passing the therapeutic window of 3 to 4 days since onset of her symptoms. However, topical steroids were prescribed to prevent further eye involvement.

The exact etiology of our patient's rash is unclear, but the temporal association of initiation of a new CBD oil and the onset of the rash is suggestive of association. There is an association between SJS and meloxicam which our patient had been taking. However, her chronic use of this medication (over 5 years) suggests that it is an unlikely trigger. Reports of systemic cannabis/cannabidiol hypersensitivity reaction are extremely rare. However, reports of marijuana associated with skin reactions such as allergic reactions, acute urticaria, and cannabis arteritis—a necrotic process presenting with ulceration of the lower extremities in the setting of chronic use [10]. There has been one case report of acute generalized exanthematous pustulosis (AGEP) in the setting of CBD use which is a type IV hypersensitivity similar to SJS-TEN [11]. In our case, the patient has previously utilized other commercial CBD products without side effects or associated allergic reactions. This suggests involvement of other ingredients in this non-FDA-regulated product as the causative agent. The complete chemical analysis from the patient's commercial CBD oil was not performed. In addition, our case also had minimal ocular involvement compared to other mucocutaneous surfaces.

We presented a case of a patient with CBD oil-induced SJS-TEN. Progression of ophthalmic involvement was prevented with cessation of the CBD oil product and initiation of an aggressive lubrication and topical anti-inflammatory regimen. It is unclear if marijuana-derived/CBD products can induce SJS-TEN. However, our case suggests an association. Further research and community surveillance is required to determine the risks of SJS-TEN from CBD products, especially given the severity and even potential fatality of this disease. Given the increased availability and use of commercial CBD products, clinicians should be aware of the possible association of CBD and SJS-TEN.
